# Feature attention graph neural network for estimating brain age and identifying important neural connections in mouse models of genetic risk for Alzheimer’s disease

**DOI:** 10.1162/imag_a_00245

**Published:** 2024-07-31

**Authors:** Hae Sol Moon, Ali Mahzarnia, Jacques Stout, Robert J. Anderson, Zay Yar Han, Jessica T. Tremblay, Cristian T. Badea, Alexandra Badea

**Affiliations:** Department of Biomedical Engineering, Duke University, Durham, NC, United States; Quantitative Imaging and Analysis Laboratory, Department of Radiology, Duke University School of Medicine, Durham, NC, United States; Brain Imaging and Analysis Center, Duke University School of Medicine, Durham, NC, United States; Department of Neurology, Duke University School of Medicine, Durham, NC, United States

**Keywords:** Alzheimer’s disease, aging, brain age, graph neural network, diffusion MRI, brain connectomics, APOE

## Abstract

Alzheimer’s disease (AD), a widely studied neurodegenerative disorder, poses significant research challenges due to its high prevalence and complex etiology. Age, a critical risk factor for AD, is typically assessed by comparing physiological and estimated brain ages. This study utilizes mouse models expressing human alleles of APOE and human nitric oxide synthase 2 (*hNOS2*), replicating genetic risks for AD alongside a human-like immune response. We developed a multivariate model that incorporates brain structural connectomes, APOE genotypes, demographic traits (age and sex), environmental factors such as diet, and behavioral data to estimate brain age. Our methodology employs a Feature Attention Graph Neural Network (FAGNN) to integrate these diverse datasets. Behavioral data are processed using a 2D convolutional neural network (CNN), demographic traits via a 1D CNN, and brain connectomes through a graph neural network equipped with a quadrant attention module that accentuates critical neural connections. The FAGNN model demonstrated a mean absolute error in age prediction of 31.85 days and a root mean squared error of 41.84 days, significantly outperforming simpler models. Our analysis further focused on the brain age delta, which assesses accelerated or delayed aging by comparing brain age, predicted by FAGNN, to the chronological age. A high-fat diet and the presence of the human*NOS2*gene were identified as significant accelerators of brain aging in the old age group. Key neural connections identified by FAGNN, such as those between the cingulum, corpus callosum, striatum, hippocampus, thalamus, hypothalamus, cerebellum, and piriform cortex, were found to be significant in the aging process. Validation using diffusion MRI-based metrics, including fractional anisotropy and return-to-origin probability measures across these connections, revealed significant age-related differences. These findings suggest that white matter degradation in the connections highlighted by FAGNN plays a key role in aging. Our findings suggest that the complex interplay of APOE genotype with sex, immunity, and environmental factors modulates brain aging and enhance our understanding of AD risk in mouse models of aging.

## 1 Introduction

Alzheimer’s disease (AD) is a devastating and irreversible neurodegenerative disorder, affecting millions of people worldwide and posing significant challenges to healthcare systems and society (2023 Alzheimer’s Disease Facts and Figures, 2023). The multifactorial etiology of AD, which encompasses genetic, environmental, and lifestyle factors, has made the comprehensive understanding of it complex but essential (Holtzman et al., 2011). Investigating the underlying risk factors associated with AD and their interactions has become a crucial task in neuroscience research (Jack et al., 2018). One of the promising methodologies has been the study of brain connectivity through magnetic resonance imaging (MRI) because of its potential to enhance our understanding of brain networks and their vulnerability, in association with cognition (Le Bihan et al., 2001). Tractography based on diffusion MRI allows construction of structural connectomes (Calabrese et al., 2015;Hagmann et al., 2008;Jones et al., 2013), and modeling properties of the connections between brain regions. Mouse models, given their genetic modifiability, are highly effective research tools. They enable detailed, high-resolution ex vivo MRI analysis (Badea & Johnson, 2012), which helps in mapping neural circuitry and identifying potential abnormalities in AD models (Badea et al., 2010,^2019^;Badea, Stout, et al., 2022;Daianu et al., 2015;Kerbler et al., 2013;Van der Linden & Hoehn, 2022;Zerbi et al., 2013).

The heterogeneity of lifestyle experience, environmental influences, and genetic traits leads to complex and multifaceted neurobiological changes during aging, and some of these changes can increase the risk for neurodegenerative conditions such as AD (Fjell et al., 2014). Age is the most significant risk factor for developing AD, and brain pathology can manifest decades before a clinical diagnosis is made (Sperling et al., 2011). Thus, understanding age-related changes in the brain is imperative. Neuroimaging-based models hold significant potential, not just as diagnostic or predictive tools, but also as markers of disease progression, patient stratification, and the effectiveness of therapeutic interventions (Cole et al., 2017;Franke et al., 2010). The disparity between an individual’s brain age and their chronological age can serve as a metric to ascertain AD risk, and to locate vulnerable subnetworks, which in turn can guide targeted early interventions (Gaser et al., 2013;Johnson et al., 2018).

Preclinical mouse models are pivotal in AD research. They allow for the controlled integration of genetic and environmental factors, managing the complex heterogeneity of AD risk elements (Zhong et al., 2024). Unlike humans, the relatively short lifespan of mice—approximately 2 years—facilitates the study of aging, a predominant factor in AD (Zhong et al., 2024). While the aging effect on the brain of the APOE genotypes in mice may differ from that of humans, incorporating environmental factors such as diet can exacerbate cognitive decline (Yassine & Finch, 2020;Zhang et al., 2024). In our study, we used mouse models with homozygous human APOE2, APOE3, and APOE4 genotypes. This approach helps address challenges in human studies, where heterozygous APOE alleles can obscure the specific impacts of each genotype (Foley et al., 2022;Williams et al., 2023).

Furthermore, we replaced the mouse*Nos2*gene with the human*NOS2*gene (*HuNOS2tg*/*mNos*-/-) to address differences in inflammatory responses between the species (Hoos et al., 2014). Humans typically express lower levels of*NOS2*and produce less nitric oxide (NO) in response to inflammatory stimuli compared with mice (Hoos et al., 2014). By introducing the human*NOS2*gene, we adjusted the redox balance which is crucial in AD progression (Vitek et al., 2020). The removal of the*mNos2*gene and the introduction of the human counterpart reduces NO production, aligning mouse immune and redox activities more closely with those observed in humans, which is linked to advancing multiple AD pathologies (Vitek et al., 2006). In this study, mice with the three major human APOE alleles and the human*NOS2*gene were fed a high-fat diet (HFD) and their outcomes were compared with nonmodified counterparts.

Statistical methods from regression to support vector machines and Gaussian processes have been employed to predict disease conversion, e.g., from MCI to AD (Gaser et al., 2013), and more recently for brain age prediction (Irimia et al., 2015), with a great shift toward deep learning approaches in recent years (Feng et al., 2020), in particular based on single (Cole et al., 2017), and also multiple imaging protocols (He et al., 2021;Liem et al., 2017). Global and local transformers have been employed to make use of global and local information (He et al., 2022). New GPU architectures and large public MRI databases have contributed to an accelerated development of image-based models to classify subjects in disease groups, predict age (Antipov et al., 2017;Cole et al., 2017;Jonsson et al., 2019;Peng et al., 2021;J. Wang et al., 2019), segment lesions (Moon et al., 2022), etc.

Brain age prediction models seek to estimate the biological age of the brain using various imaging data and have become a crucial tool in understanding aging and neurodegeneration. Deep learning models, particularly those employing convolutional neural networks (CNNs) and graph neural networks (GNNs), have shown significant promise in capturing complex patterns in brain imaging data (Azevedo et al., 2022;Zhao et al., 2022). These models often use large datasets to train on features extracted from MRI, fMRI, or PET scans, providing a prediction of brain age that can then be compared with chronological age to assess anomalies (Nguyen et al., 2024;Smith et al., 2019). The discrepancy between predicted brain age and chronological age can offer insights into the patient’s neurological health, where a greater disparity may indicate the presence or risk of neurodegenerative diseases (Wein et al., 2021).

CNNs have been particularly pervasive due to their effectiveness in handling high-dimensional data such as fMRI (Kaufmann et al., 2019). GNNs were found to be well suited for dealing with fMRI-derived connectomes (Ktena et al., 2017;Li et al., 2021) and structural connectivity (Lin et al., 2016), which can benefit from multimodal integration with structural MRI using graph transformers (Cai et al., 2023). Such models may benefit from age-specific stratification, and from integration of external data, allowing one to examine the relationship between cognition and brain imaging-derived estimates (Lee, 2023;Millar et al., 2023). Yet, despite these advances, diffusion MRI, with its ability to provide brain structural connectivity, has yet to be fully explored using neural networks for tasks such as age prediction (Bass et al., 2023;Vakli et al., 2018).

In previous connectome-based prediction studies, the focus has been on brain regions, which make up the nodes in a connectome, but understanding AD’s progression should address changes in the edges connecting such nodes (Hagmann et al., 2008). In order to discern the important connections, there is a need to minimize connectomic noise and mitigate potential bias and overfitting due to the immense number of edges present in a connectome (Anderson et al., 2019;Calabrese et al., 2015). Further, the small sample sizes and data heterogeneity from genetic and environmental factors from our mouse models compound these challenges (Bettio et al., 2017;Liu et al., 2015).

To address these issues and achieve our objective of predicting brain age in a mouse population, we propose a Feature Attention Graph Neural Network (FAGNN) model. The proposed FAGNN’s architecture utilizes distinct subnetworks tailored to process each data type, encompassing connectomes, risk factors, and behavioral data, thereby ensuring a more robust and comprehensive analysis (Erwin et al., 2014;Raj et al., 2010;Smith & Topin, 2019). Drawing inspiration from the multihead attention mechanisms of transformer models (Vaswani et al., 2017), we developed a quadrant attention module (QAM) to capture the interplay of brain connections more efficiently by processing each quadrant of the connectome separately as feature dimensions. This approach enables the QAM within FAGNN to efficiently capture and highlight task-specific brain connections by leveraging unique data from each connectome quadrant for a comprehensive analysis. Unlike previous GNN architectures that emphasize important nodes, FAGNN highlights connections based on edges (Gao & Ji, 2019;Li et al., 2021).

In our study, we aimed to validate the network’s identification of important structural connections by examining tractography features that may influence the structural connectome. We initially utilized diffusion weighted imaging (DWI) metrics, specifically fractional anisotropy (FA), to assess the directional diffusion of water molecules within brain tissues. FA provides valuable insights into the microstructural integrity of white matter tracts (Murphy & Frodl, 2011;Sullivan & Pfefferbaum, 2006). However, this model is based on the assumption of Gaussian water diffusion, which may not adequately represent the complexity of brain tissue microstructures. To address the non-Gaussian nature of water diffusion in the brain, particularly in regions with intricate fiber configurations, we incorporated Mean Apparent Propagator (MAP) metrics, derived from models such as the Simple Harmonic Oscillator-based Reconstruction and Estimation (SHORE), which may provide a more precise representation of the diffusion process in brain tissues (Özarslan et al., 2013). These metrics include return-to-origin probability (RTOP), return-to-axis probability (RTAP), and return-to-plane probability (RTPP), which correspond to various aspects of pore structure as described in pore theory (Callaghan et al., 1992). Specifically, RTOP is associated with the average volume of pores, RTAP to their cross-sectional area and RTPP to their length as detailed in earlier studies, meaning higher values of these metrics reflect a more restricted diffusion in neural tissues (Bogusz et al., 2022;Mitra et al., 1995;Özarslan et al., 2013). These quantitative indices offer valuable information of tissue heterogeneity (Brusini et al., 2018), ischemic stroke (Brusini et al., 2016;Galazzo et al., 2018), multiple sclerosis (Brusini et al., 2021), cognitive impairment detection (Menon et al., 2020;Pitteri et al., 2021), and aging and dementia (Bouhrara et al., 2023).

The primary objective of this study was to explore the potential of advanced neural network architectures, specifically the FAGNN, for predicting brain age, as age is the most significant risk factor for AD. By utilizing a deep learning-based integrative modeling approach that incorporates multivariate datasets including brain connectomes, traits such as sex and genotypes, and cognitive metrics, we aim to reveal the interactions between such factors and the aging process. We sought to identify specific brain connections that play a significant role in age prediction, offering deeper insights into possible association with the progression of brain aging. Our approach can serve to find early biomarkers related to aging, to facilitate the design and testing of future preventative and therapeutic interventions.

## 2 Methods

### 2.1 Animal models

In this study, we examined the effects of five contributing factors on AD risk within brain connectomes: APOE genotype, presence of human*NOS2*gene (HN), diet, age, and sex. We utilized animal models in which the mouse APOE gene was replaced with the three major human APOE variants. These mice were homozygous for the APOE2, APOE3, and APOE4 alleles. To better reflect the human innate immune system, we included mice with a human*NOS2*gene (Colton et al., 2008). To address sex as a biological variable, and increased risk for AD, our sample selection included male and female mice. In our study of age-related risk factors, we included a total of 170 mice separated into two cohorts: a group of 66 mice aged 12 to 15 months to represent middle age, and a group of 104 mice aged 15 to 20 months to represent old age. To introduce an environmental risk factor, we subjected mice to either a standard or high-fat diet. The animal groups were distributed across APOE genotypes (58 APOE2, 56 APOE3, and 56 APOE4 homozygous mice), sex (82 males and 88 females), HN (95 mice with mouse*mNos2*and 75 mice with human*hNOS2*), and diet (113 control diet and 57 high-fat diet).Table 1displays the distribution of animals used in this study. The use of these mice and the experiments conducted were approved by the Duke IACUC (Institutional Animal Care and Use Committee).

**Table 1.** Distribution of experimental mouse cohorts by age, APOE genotype, sex,*NOS2*gene, and diet type.

Age cohort	Total mice	APOE genotype			Diet		*NOS2* gene		Sex	
		APOE2	APOE3	APOE4	High-fat diet	Control diet	Mouse *mNos2*	Human *hNOS2*	Male	Female
12-15	66	17	24	25	33	33	24	38	32	34
15-20	104	41	32	31	24	80	67	37	50	54

The table outlines the cohort composition used to investigate the combined effects of genetic background, sex, and diet on aging. Mice aged 12-15 months represent younger age, while mice aged 15-20 months represent old age. The mice are further categorized by APOE genotype (APOE2, APOE3, APOE4), sex (male, female),*NOS2*gene (*mNos2*or*hNOS2*), and diet (control or high fat) to assess the influence of these variables on the progression of age-related changes.

### 2.2 Image acquisition and connectomes

To derive structural connectomes, we used high-field, 9.4T MRI to image fixed brain specimens, using a previously published protocol (Badea, Li, et al., 2022;Winter et al., 2023). To prepare the brain specimens for imaging, mice were sacrificed through a transcardiac perfusion fixation with 10% formalin and 10% (50 mMol) gadoteridol (ProHance, Bracco), then the mouse brains were trimmed and immersed in 0.05% (2.5 mMol) ProHance with PBS (0.01 molar, pH 7.4). A compressed sensing 3D SE sequence was used for DWI, with the following parameters: TR/TE of 100 ms/14.2 ms, and BW of 62.5 kHz. Diffusion pulses were applied in 46 directions with 2 diffusion shells (23 at 2,000 and 23 at 4,000 s/mm²) and we used 5 nondiffusion-weighted (b0) images. The diffusion pulse had a maximum amplitude of 130.57 Gauss/cm, a duration of 4 ms, and a separation of 6 ms, with 8-fold compressed sensing acceleration (Anderson et al., 2019;N. Wang et al., 2018). A matrix size of 420 x 256 x 256 and FOV of 18.9 mm x 11.5 mm x 11.5 mm produce images with 45 μm isotropic resolution. Tractography was conducted utilizing a Q-ball algorithm with an FA threshold of 0.07 and a tract deviation angle limited to 60°. We used MRtrix3 (Tournier et al., 2019) to create tractography and connectomes based on the acquired diffusion MRI images. Approximately 10 million tracts were generated, from which a subset of 2 million tracts was selected for each subject, based on an FA cutoff of 0.1. Computational and storage constraints necessitated the downsampling of our dataset from 10 million to 2 million streamlines, ensuring a minimum streamline length of 100 μm to mitigate the inclusion of spuriously short tracts. We calculated the diffusion-based microstructural parameters using MRtrix3 (Tournier et al., 2019), and RTOP, RTAP, and RTPP using Dipy (Garyfallidis et al., 2014).

### 2.3 Behavioral metrics

Mice with genetic and environmental risk factors for AD can exhibit cognitive decline, including learning and memory deficits, which are characteristic of both aging and AD. To quantitatively assess behavioral differences, we utilized the Morris Water Maze (MWM) test. Mice were introduced to a pool filled with room temperature water, rendered opaque by the addition of nontoxic white paint. The pool was divided into four quadrants (SW, NE, NW, SE), a thigmotaxis area, and a hidden platform located in the SW quadrant’s center. During training trials, the mice were released from each quadrant for four trials per day for 5 consecutive days and had to find the hidden, submerged platform using external visual cues, swimming for a maximum of 60 seconds. We measured the time and distance to platform and quadrant-specific measures to evaluate spatial learning and memory. Two probe tests were conducted: at 1 hour after the final training trial (day 5), and another one at 72 hours (day 8) to evaluate memory retention for the platform location. Metrics included swim distance, swim velocity, time to locate platforms, time and distance in each quadrant, number of entries to probe location, and winding numbers representing rotational movement during swimming (Badea, Li, et al., 2022).

### 2.4 Feature attention graph neural network (FAGNN)

Binary risk factors such as sex, diet, and*NOS2*gene were normalized to values of 0 or 1. The genotype data of APOE alleles (2, 3, and 4) were scaled to 0, 0.5, and 1, respectively. We ranked normalized connectome data, representing the connections between different brain regions. Equal number of connections were assigned the same rank, which was then divided by the maximum rank, and scaled between 0 and 1. Behavioral data were also normalized, with time-based metrics divided by 60, which is the maximum time duration (in seconds) of each MWM trial, and other metrics divided by their corresponding maximum values to bring them to a similar scale. Thus, the entire data used in the network were scaled between 0 and 1.

**Fig. 1.**
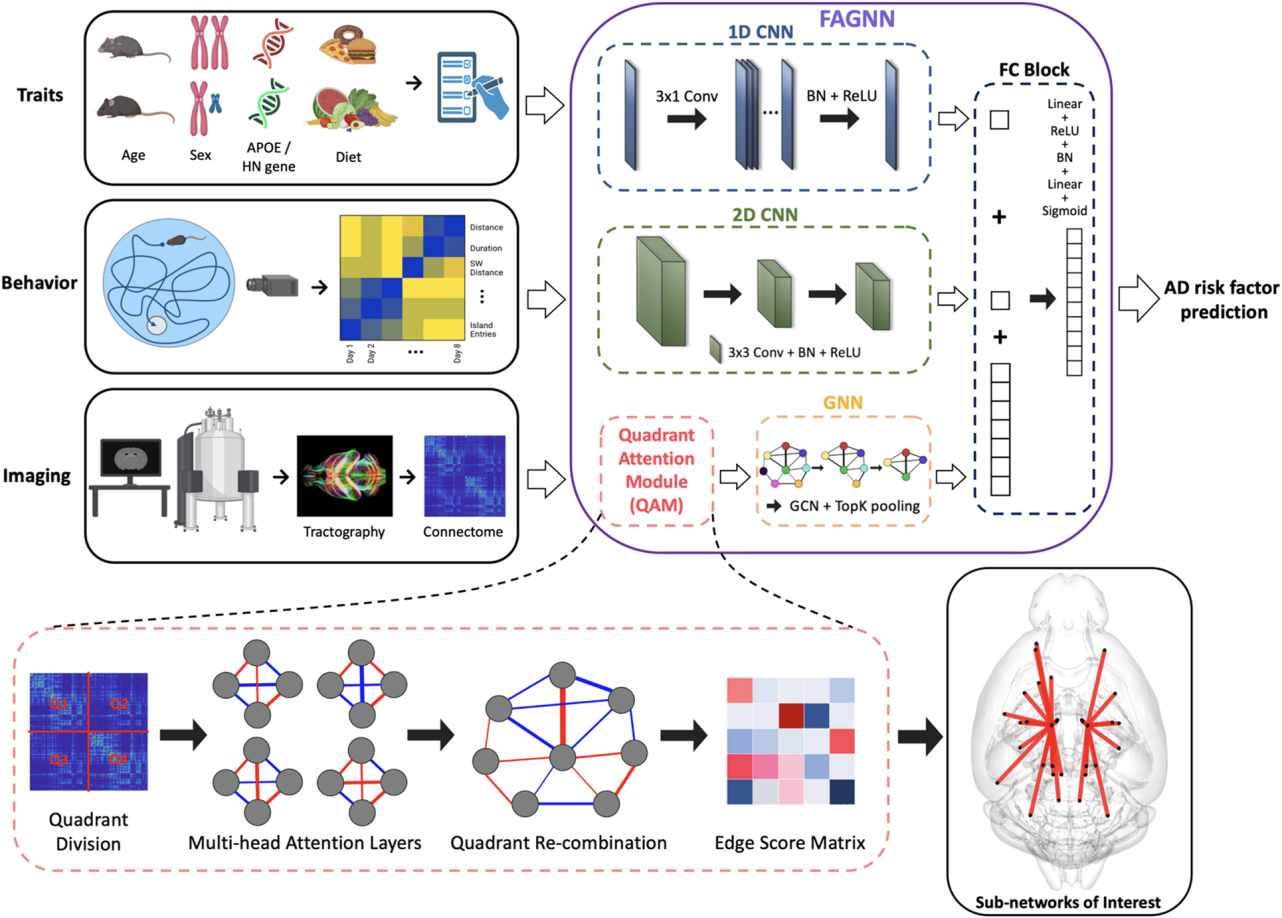
Overview of FAGNN method integrating AD risk factor traits, behavioral metrics, and brain connectome. Diffusion MRI-derived connectomes undergo quadrant attention module for edge scoring prior to GNN analysis. AD risk traits and behavioral metrics are processed by 1D and 2D CNNs, respectively. The network outputs predicted brain age and we assess the difference between brain age and chronological age as a risk factor.

Our integrative modeling approach for age prediction (Fig. 1) includes multiple optimized subnetworks for each input modality. The behavioral data are fed through a 2D CNN. The arrangement of the behavioral data ensures that similar metrics placed adjacently in columns and trials progressing from earlier to later are represented in a spatially contiguous manner, which aids the 2D CNN in effectively extracting spatial features and patterns that are relevant to the progression of the behavioral data. Risk factor data based on subject traits (diet, sex, genotype), being one-dimensional, were passed through a 1D CNN. This is effective for capturing patterns across the 1D data especially given that we later combine these output tensors with those of 2D CNN for tensor homogeneity. This process captures cognitive markers associated with disease biomarkers. Connectome data are first passed through a quadrant attention module (QAM), which is responsible for assigning important scores to different brain connections. This helps in highlighting the most important connections in the brain with respect to AD risk factors. After the QAM, the connectome graph data were multiplied elementwise with the edge scores, then processed by a GNN, which incorporated Graph Convolutional Networks (GCNs) and top-K pooling layers (Gao & Ji, 2019). GNNs are particularly effective for this study because the brain connectome represents a complete graph structure, where each node or brain region is connected to every other node, thereby maximizing the potential for graph learning (Bullmore & Bassett, 2011;Scarselli et al., 2009). GNNs have also demonstrated their capability in processing unique graph structures such as chemical compounds, proteins, or maps, where a complete graph structure offers extensive information for each data point (Du et al., 2022;Kojima et al., 2020).

Previous studies, such as those by Li et al., have often focused on identifying important nodes using a top-K scoring mechanism primarily for noise reduction, rather than for accurately pinpointing critically important brain regions (Gao & Ji, 2019;Li et al., 2021). In contrast, our method preserves all available edges, assigns scores to each, and provides a comprehensive view of the entire network, rather than merely focusing on individual nodes. Additionally, our methodology utilizes diffusion MRI, distinct from the fMRI-based techniques employed in the original BrainGNN model (Li et al., 2021). Diffusion MRI measures direct connections through water diffusivity, emphasizing the significance of distances between connections. Consequently, incorporating a QAM that processes hemispheric connections separately enhances the relevance of connectivity analyses.

We then process the connectome using the GNN subnetwork based on BrainGNN (Li et al., 2021) that is designed for classification tasks. We modified the model by including a mean-squared error loss function to be able to predict age, which is a continuous variable. The top-K pooling layers within the GNN are instrumental in selecting the top-K percent of nodes in each graph layer, prioritizing nodes based on their importance for reducing noise and decreasing dimensionality. In our model, the top-K pooling is employed solely for processing the connectome in age prediction and is not utilized for identifying node importance for attention purposes. The output tensors from each subnetwork were then concatenated and passed through a fully connected (FC) block, comprising multiple layers of a multilayer perceptron (MLP).

In QAM, we utilize the structure of brain connectome data, organized as an*n*×*n*matrix that illustrates the interconnections between*n*different brain regions. To effectively manage the high dimensionality and potential symmetry within the connectome, we divide the matrix into four quadrants corresponding to left-left, left-right, right-left, and right-right hemispheric connections. Each quadrant is separately processed by a multihead attention (MHA) mechanism, generating specific edge scores for each quadrant. These scores from the four quadrants are then combined to form a complete set of edge scores for the entire connectome.

The MHA mechanism incorporates the scaled dot-product attention mechanism, which is primarily utilized in transformers as proposed byVaswani et al. (2017). This approach has demonstrated effectiveness in processing complex patterns within natural language processing (NLP). In our study, we derived a simpler variant of this attention mechanism from the transformer architecture. Unlike the module used in NLP, which predicts the next token, our attention mechanism focuses on calculating edge scores which are then used to obtain brain age prediction. These scores reflect the importance of connections within the connectome, which are subsequently analyzed by a GNN for age prediction.

Given an input connectome data tensor$X\in {\mathbb{R}}^{n}\times {\mathbb{R}}^{n}$, we partition it into four quadrants:

$$X=\left[\begin{array}{cc} X_{LL} & X_{RL}\\ X_{LR} & X_{RR} \end{array}\right]$$

$$X_{LL}=X\left[\alpha ,\alpha \right],\;\;X_{LR}=X\left[\alpha ,\beta \right],\;\;X_{RL}=X\left[\beta ,\alpha \right],X_{RR}=X\left[\beta ,\beta \right]$$

$$\alpha =\left(1,\ldots ,\frac{n}{2}\right),\beta =\left(\frac{n}{2}+1,\ldots ,n\right)$$.

Each quadrant$X_{pq}$, with*p*,*q*is then processed through MHA separately. The MHA operation is formulated as follows (Vaswani et al., 2017):

$$MHA(X_{pq})=Concat\left(head_{1},\ldots ,head_{h}\right)W^{O}.$$

Here, each attention head$head_{j}$forj= 1,…,his computed using the scaled dot-product attention mechanism:

$$head_{j}=Attention\left(X_{pq}W_{j}^{Q},X_{pq}W_{j}^{K},X_{pq}W_{j}^{V}\right),$$

where$W_{j}^{Q}$,$W_{j}^{K},$and$W_{j}^{V}$are the weight matrices for the query (Q), key (K), and value (V), respectively, for$head_{j}$, and$W^{O}$is the output weight matrix that combines the heads. The attention function is defined as

$$Attention\left(Q,K,V\right)=softmax\left(\frac{QK^{T}}{\sqrt{d_{k}}}\right)V,$$

where$d_{k}$is the dimension of each key. We concatenate the edge scores of each quadrant back to shape of the original connectome as follows:

$$S=\left[\begin{array}{cc} MHA\left(X_{LL}\right) & MHA\left(X_{LR}\right)\\ MHA\left(X_{RL}\right) & MHA\left(X_{RR}\right) \end{array}\right],$$

where*S*is the edge score matrix. In the QAM, this scaled dot-product attention mechanism is applied separately to each quadrant of the connectome. The four quadrants correspond to the brain’s hemispheric divisions: left-to-left (LL), right-to-right (RR), left-to-right (LR), and right-to-left (RL) hemispheric connections. This quadrant-specific processing aligns with the brain’s method of regional communication through inter- and intrahemispheric connections. Thus, the QAM in our model narrows the focus to distinct quadrants for a more targeted analysis of age-related neural connections. By utilizing MHA layers for each quadrant, QAM facilitates a detailed yet efficient edge score calculation of the connectome.

We propose a parallel subnetwork schematics for the FAGNN architecture, combining a GNN subnetwork and two CNN subnetworks. Each layer of these subnetworks is updated as follows:

HG(l+1)=ReLU[(In(l)+HG(l)⊙Sδl=1+HG(l)δl>1)D(l)WG(l)]

HC1(l+1)=ReLU[WC1(l)HC1(l)+bC1(l)]

HC2(l+1)=ReLU[WC2(l)HC2(l)+bC2(l)].

We setHm(0)=Xmas the input in the first layer, where*m*can beGfor GNN,$C_{1}$for 1D CNN, and$C_{2}$for 2D CNN. The input to the GNN is the structural connectome, followed by element-wise multiplication of edge scores*S*derived from the QAM, as denoted by the delta function. The input to 1D CNN is trait information, and the input to 2D CNN is behavior. Here,lis the current level of layer in the network,$I_{n^{\left(l\right)}}$is the identity matrix with*n*nodes at layerl,$D^{\left(l\right)}$is the diagonal matrix of the graph data at layer*l*,Wm(l)andbm(l)are the learnable model parameters and the bias of subnetwork*m*at layer*l*, respectively.

The$NL_{G}$,$NL_{C_{1}}$, and$NL_{C_{2}}$are the final layer numbers for each subnetwork, respectively, before the MLP. The outputs of the last layer of each subnetwork are concatenated and passed through MLP as follows:

y^=MLP[HGNLGHC1NLC1HC2NLC2],

where$\hat{y}$represents the brain age predicted by the FAGNN model.

After the QAM, the FAGNN is employed to process the connectomic graph data. FAGNN integrates GCNs and CNNs to perform nonlinear transformations, computing the subsequent hidden state$H^{\left(l+1\right)}$based on the output from the quadrant attention moduleS. This enables FAGNN to efficiently represent the graph structure of connectome, while accounting for the attributes of nodes and edges within the graph. Moreover, given that the dataset encompasses mice exhibiting multiple risk factors, the inclusion of risk factor data in the prediction helps the model to discern and account for the potential influence of various risk factors and their intricate interplay on brain connectivity, ensuring a less biased representation. Additionally, the incorporation of behavioral data is beneficial as it represents cognitive functions, enabling the model to account for potential correlations with brain connectivity patterns and AD risk factors. By integrating these elements, FAGNN contributes to a more holistic comprehension of the interplay between brain connectivity, cognitive performance, and AD risk factors. Additionally, we utilized a modified Adam Optimizer with distinct learning rates for each subnetwork to accommodate the varying rates of learning convergence associated with the different types of neural networks used in this study.

To train the model efficiently, we used different learning rates for different subnetworks, which is helpful considering the different types of data being processed by each subnetwork. The optimizer computes the moving averages of gradients and their squares and uses them to adaptively adjust the learning rates during training. This helps in achieving faster convergence during training and can lead to better performance of the model. Each subnetwork was assigned its unique learning rate depending on the performance. The full model integrates subnetworks including QAM, GNN, 1D CNN, 2D CNN, and FC block. The respective learning rates for these subnetworks were 0.001, 0.001, 0.002, 0.002, and 0.003 with 100 epochs.

In our model evaluation, we utilized a fivefold cross-validation approach, where the dataset was divided into five distinct folds for comprehensive training and testing. This methodology allowed us to compare the performance of the FAGNN model against several variations: Comp 1 utilized connectome data processed via a modified BrainGNN, Comp 2 combined the QAM with the connectome, Comp 3 added behavioral data to the QAM and connectome, Comp 4 integrated trait data with the QAM and connectome, and the full FAGNN model included the QAM, connectome, trait data, and behavioral data. The accuracy of these models was quantified by calculating the mean absolute error (MAE) and root-mean-squared error (RMSE). Each model was run 10 times using the full dataset and 20 times when training different groups separately to ensure consistency in the results. For our statistical analyses, we employed linear models within the R programming framework, considering a*p*-value less than 0.05 to be statistically significant.

Linear mixed models were used for tract-based analysis of FA and MAP parameters. Post hoc pairwise comparisons were conducted using Dunn’s test to calculate the significance of difference of each group separately after a nonparametric Kruskal–Wallis test. Considering the multiple comparisons being made,*p*-values from Dunn’s test were adjusted using the Holm method to control the familywise error rate. We trained the models using four RTX 6000 cards and two RTX 8000 cards, each with 48 GB of memory. The time to train the model on entire dataset (N = 170) was 5.2 minutes with 100 epochs using a single Nvidia RTX 6000 GPU. The results were visualized using DSI studio (http://dsi-studio.labsolver.org) and brainconn2 (Mahzarnia et al., 2023).

## 3 Results

To help estimate risk for AD based on the discrepancy between chronological and brain age, we built a brain age prediction model using a dataset of 170 mice. Although this represents one of the largest published mouse studies, it remains limited in sample size compared with human studies, which typically feature greater genetic and environmental exposure variability. Our strategy used 5-fold cross-testing, with each fold including 136 mice used for training and 34 mice used for testing, and each fold was trained for 10 runs when including full dataset and 20 runs when training on separate groups. Along with age prediction, FAGNN results included important edges and nodes. We validated these connections through a comparison of diffusion parameters using FA and MAP metrics associated with tractography, to test whether microstructural parameters support the connectivity changes during aging.

### 3.1 Model performance in age prediction

The Feature Attention Graph Neural Network (FAGNN) was assessed for its capability to estimate brain age from structural connectome data. The model’s performance was evaluated by its MAE of 31.85 days and RMSE of 41.84 days, as shown inTable 2. The FAGNN model outperformed a modified BrainGNN and other configurations of specific data and corresponding subnetwork inclusion. The second-best model included the quadrant attention module with connectome risk factors (Comp 4), followed by a similar model which included behavior data instead of the risk factors (Comp 3). The next ranked model included only the quadrant attention module with connectome (Comp 2), and the lowest ranked model only included connectome data through a modified BrainGNN (Comp 1). These measures of accuracy indicated the FAGNN full model’s proficiency in estimating brain age, which is a conceptual age derived from brain connectome patterns, and thus may differ from chronological age. This estimation forms the basis for subsequent analyses of brain aging and the impact of various risk factors.

**Table 2.** Age prediction performance of various combinations of GNN and FAGNN with repeated 10 runs.

Method	MAE days (95% CI)	RMSE days (95% CI)
Comp 1	42.594 (40.988, 44.200)	54.363 (52.315, 56.337)
Comp 2	37.665 (36.310, 39.020)	47.231 (45.583, 48.823)
Comp 3	36.072 (34.587, 37.558)	47.720 (45.858, 49.512)
Comp 4	36.058 (34.721, 37.394)	45.717 (43.933, 47.435)
FAGNN full model	31.851 (30.561, 33.141)	41.844 (40.184, 43.440)

The testing metrics include MAE and RMSE. Comp 1: connectome (modified BrainGNN); Comp 2: QAM + connectome; Comp 3: QAM + connectome + behavior; Comp 4: QAM + connectome + risk factors. FAGNN full model included QAM + connectome + risk factors + behavior.

Due to the limited number of subjects in our dataset (N = 170), we assessed the entire dataset to determine the MAE as a measure of model performance. However, evaluating MAE for the entire dataset may not fully reflect the nuanced performance capabilities of models, particularly in detecting aging related to various factors, where a higher MAE could indicate greater sensitivity. This sensitivity is crucial for identifying changes attributable to different factors. Consequently, we conducted separate fivefold validations for all the mouse groups, as detailed inTable 3. We also calculated the delta MAE, representing the difference between the MAE of the two groups. Assuming that the group with the higher MAE is generally indicative of greater sensitivity to the factors being studied, we trained models on one group and tested them on the other. The results are shown inTable 4. The FAGNN model exhibited the lowest MAE among most groups and displayed the highest delta MAE in scenarios where the groups were trained and tested separately, as well as when trained on one group and tested on the corresponding group. This underscores FAGNN’s superior accuracy and sensitivity to the risk factors, leading to its selection for further analysis in this study.

**Table 3.** Model prediction accuracy with training and testing within the same phenotype group with repeated 20 runs.

	Models				
	Comp 1	Comp 2	Comp 3	Comp 4	FAGNN
Non-APOE4 (N = 114)	37.26 (95% CI)	39.91 (95% CI)	36.00 (95% CI)	35.24 (95% CI)	**29.50 (95% CI)**
APOE4(N = 56)	72.52 (95% CI)	**67.03 (95% CI)**	76.92 (95% CI)	74.73 (95% CI)	75.15 (95% CI)
∆ MAE APOE	35.26	27.12	40.92	39.49	**45.65**
Control diet (N = 113)	39.40 (95% CI)	39.89 (95% CI)	39.49 (95% CI)	37.66 (95% CI)	**35.07 (95% CI)**
High-fat diet (N = 57)	49.79 (95% CI)	50.67 (95% CI)	49.41 (95% CI)	49.91 (95% CI)	**49.02 (95% CI)**
∆ MAE diet	10.39	10.78	9.92	12.25	**13.95**
*hNOS2* (N = 75)	30.08 (95% CI)	33.65 (95% CI)	**25.08 (95% CI)**	33.10 (95% CI)	25.57 (95% CI)
*mNos2* (N = 95)	41.23 (95% CI)	44.33 (95% CI)	37.90 (95% CI)	38.76 (95% CI)	**36.24 (95% CI)**
∆ MAE HN	11.15	10.68	**12.82**	5.66	10.67
Female (N = 87)	37.21 (95% CI)	35.39 (95% CI)	34.51 (95% CI)	32.48 (95% CI)	**29.84 (95% CI)**
Male (N = 83)	40.96 (95% CI)	44.27 (95% CI)	43.13 (95% CI)	41.26 (95% CI)	**39.81 (95% CI)**
∆ MAE Sex	3.75	8.88	8.62	8.78	**9.97**

These include APOE genotype (non-APOE4 and APOE4), diet (control diet and high-fat diet), HN gene (human*hNOS2*and mouse*mNos2*), and sex (female and male) groups. The group that resulted in lower MAE was chosen as the training group and the group that had higher MAE was chosen as the test group based on this result. The bold values indicate the lowest group MAE values or the highest Δ MAE values when comparing two related phenotype groups.

**Table 4.** Model prediction accuracy (MAE) with trained on the train group and tested on test group for each phenotype with repeated 20 runs.

		Models				
Train set	Test set	Comp 1	Comp 2	Comp 3	Comp 4	FAGNN
Non-APOE4	APOE4	36.46 (95% CI)	35.54 (95% CI)	34.28 (95% CI)	36.51 (95% CI)	34.26 (95% CI)
∆ MAE APOE		-0.80	-4.37	-1.72	1.27	**4.76**
Control diet	High-fat diet	40.60 (95% CI)	39.52 (95% CI)	40.25 (95% CI)	38.12 (95% CI)	38.73 (95% CI)
∆ MAE diet		1.20	-0.37	0.76	0.46	**3.66**
*hNOS2*	*mNos2*	52.03 (95% CI)	53.78 (95% CI)	49.52 (95% CI)	53.78 (95% CI)	52.39 (95% CI)
∆ MAE HN		21.95	19.13	24.44	20.68	**26.82**
Female	Male	40.54 (95% CI)	44.24 (95% CI)	40.11 (95% CI)	40.98 (95% CI)	35.92 (95% CI)
∆ MAE sex		3.33	8.85	5.60	**11.14**	6.08

These include APOE genotype (trained on non-APOE4 and tested on APOE4), diet (trained on control diet and tested high-fat diet), HN gene (trained on*hNOS2*and tested on*mNos2*), and sex (trained on female and tested on male) groups. The Δ MAE for each group was calculated by subtracting mean MAE of the control group from the mean MAE of the risk factor group within each phenotype. The mean MAE of control group (fivefold cross validation) used in the calculation of ∆MAE is fromTable 3. The bold values indicate the highest Δ MAE values.

**Fig. 2.**
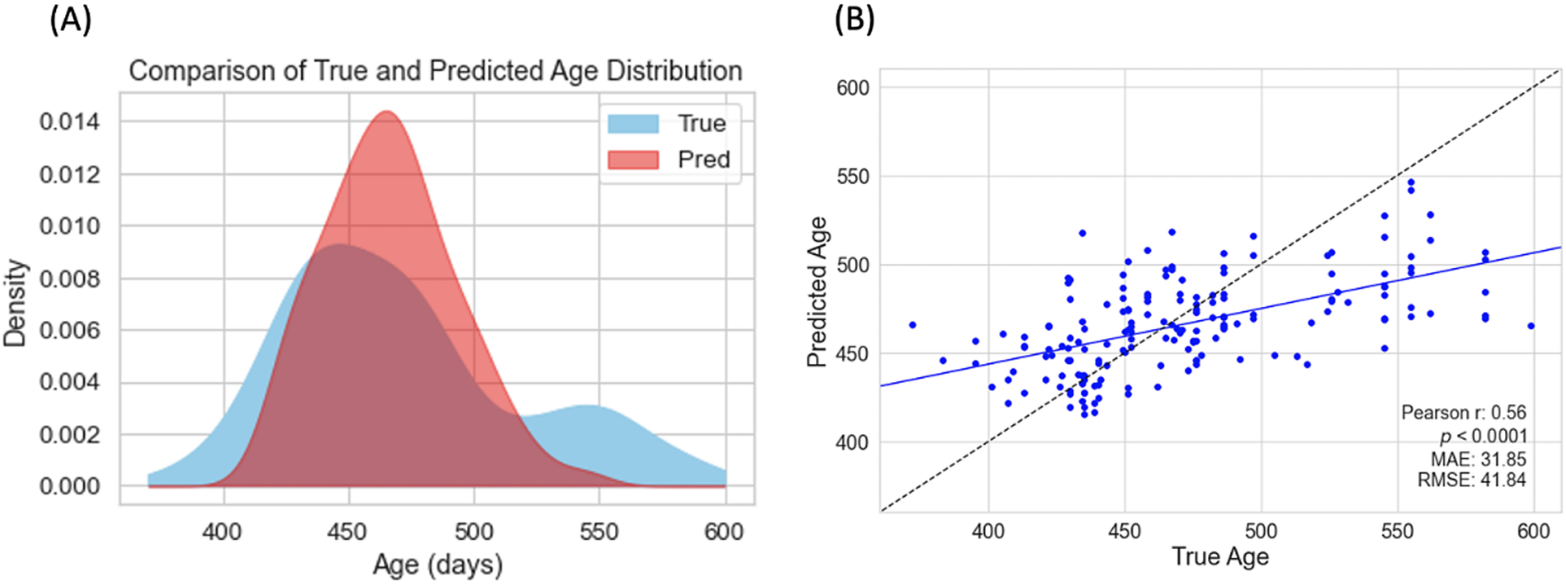
(A) Age distribution plot comparing true and predicted ages as analyzed by FAGNN. (B) Scatterplot of predicted brain age versus chronological true age using the full dataset (N = 170), evaluated with fivefold cross-validation. Each blue dot represents the predicted brain age for an individual subject, and the blue line denotes the linear regression fit.

We used smooth kernel density estimation envelope plot to provide smoothed representation of the data distribution for true and predicted age as shown inFigure 2A. The true ages display bimodal distribution characteristics, reflecting two distinct age groups within our dataset, whereas the predicted ages appear to converge into a unimodal distribution.Figure 2Billustrates the comparison of true versus predicted ages, yielding a Pearson correlation coefficient*r*of 0.56 and a highly significant*p*-value (*p*< 0.0001).

The brain age delta comparison is depicted inFigure 3. In the younger group, there were no significant differences between APOE2, 3, and 4 alleles, nor were there significant differences attributable to diet or between mouse*mNos2*and human*hNOS2*genes. However, a significant difference was observed between male and female mice (*p*< 0.05). In the old group, no significant differences were noted among APOE2, 3, and 4 alleles or between sexes. However, dietary contrasts revealed significant differences between control and high-fat diets (*p*< 0.05). Additionally, the brain age delta for those expressing the human*hNOS2*gene was significantly different from those with the mouse*mNos2*gene (*p*< 0.05).

**Fig. 3.**
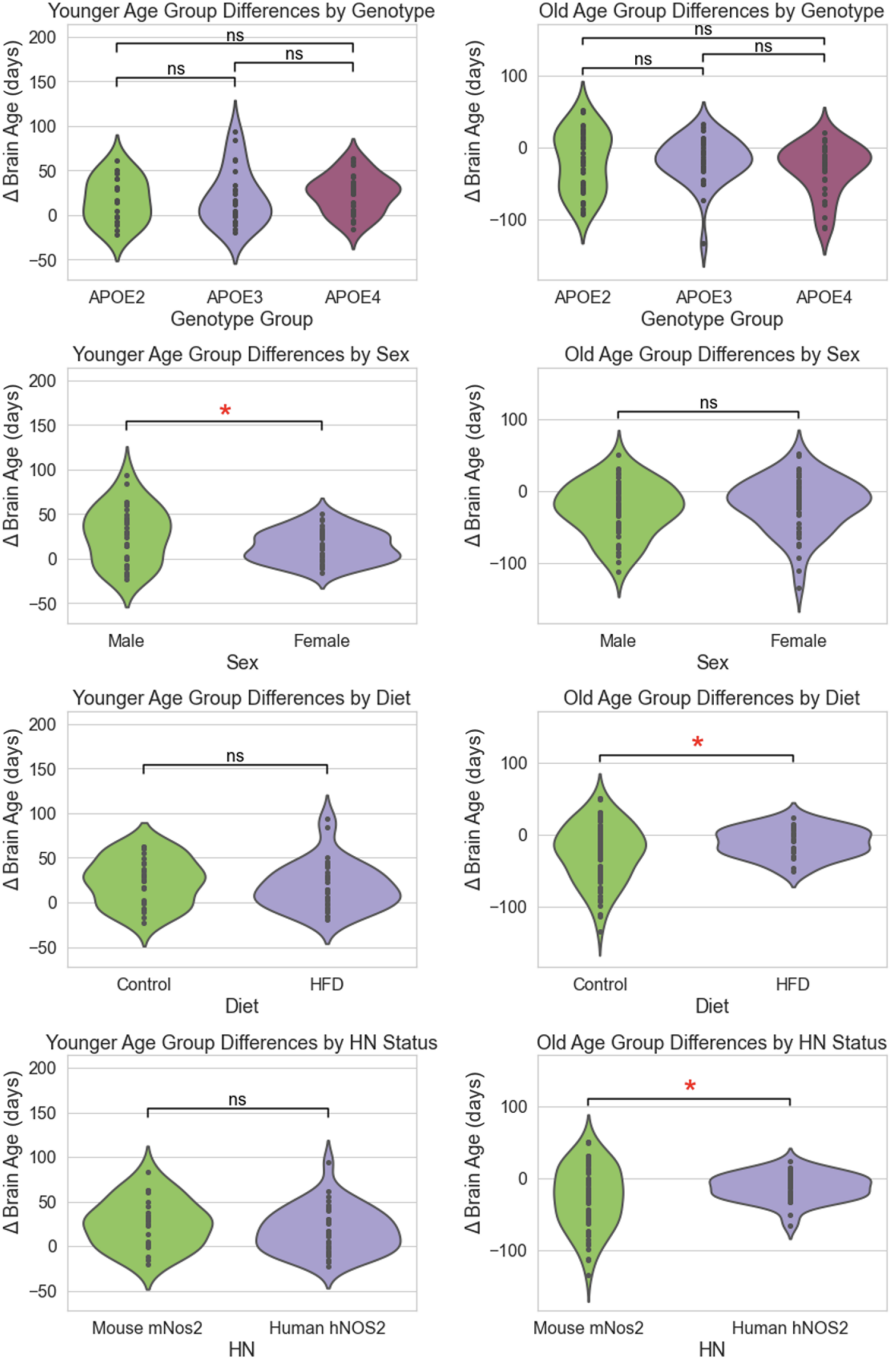
Comparison of brain age deltas between control mice and those in the risk factor group, segmented into younger and old age categories. The “*” symbol indicates significant differences with*p*< 0.05, while “ns” denotes nonsignificant*p*-values. We note that sex differences were significant in the younger mice, while diet and immunity played a larger role in the old cohorts.

### 3.2 Connectomic and tractography analysis across age groups

**Fig. 4.**
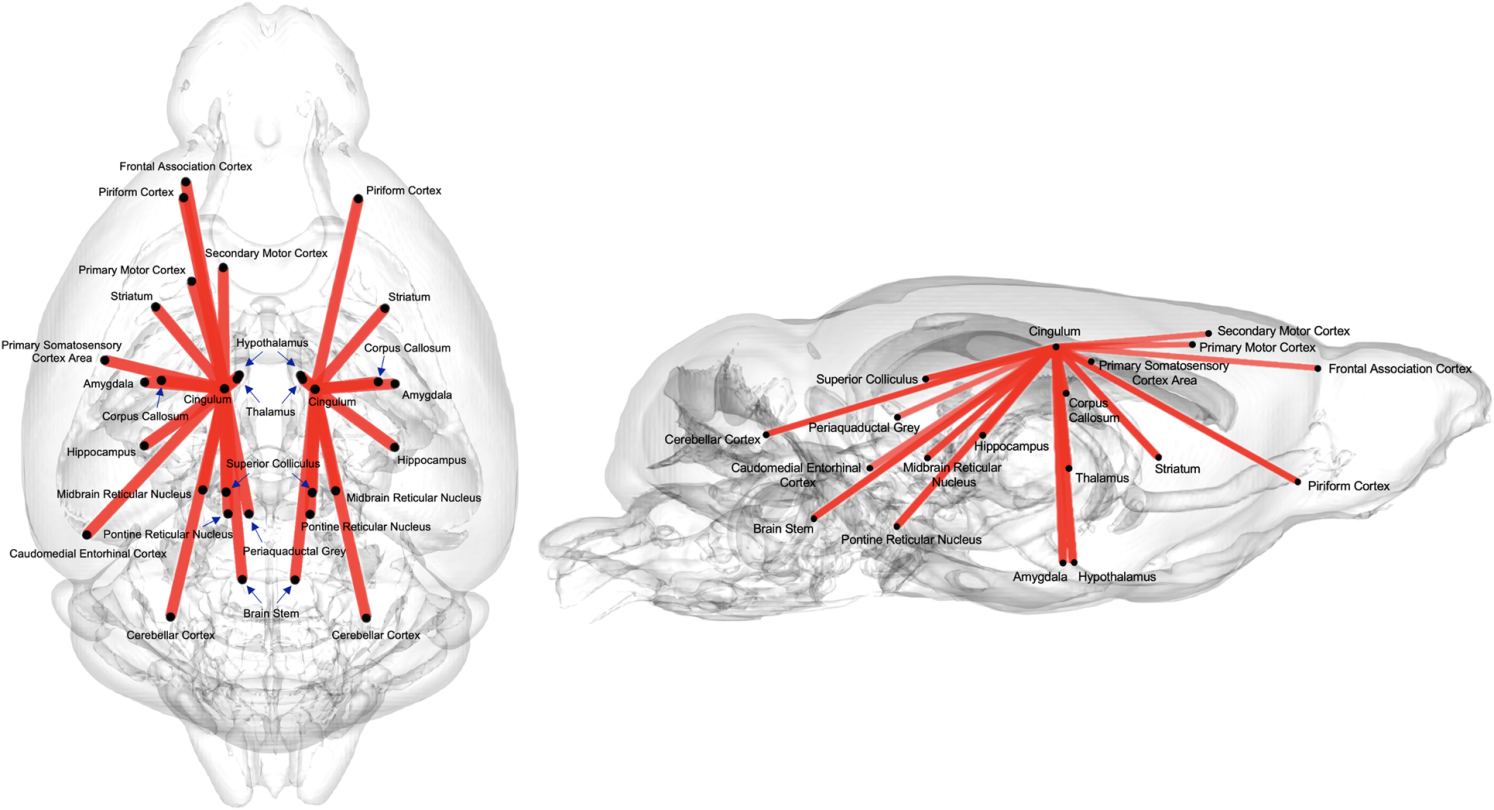
The top 30 connections with the highest edge scores from continuous age prediction, as identified by FAGNN. These connections, which show the strongest association with age variation, predominantly link to either the left or right cingulum, creating two separate but largely symmetrical subnetworks.

The FAGNN model identified the top 30 brain connections that were most influential in age prediction based on the highest weights (Fig. 4). These pointed to a central role for the cingulum and its connections. The model also highlighted several critical nodes, including the piriform cortex, frontal association cortex, primary and secondary motor cortex, striatum, amygdala, hippocampus, thalamus, midbrain reticular nucleus, pontine reticular nucleus, caudomedial entorhinal cortex, cerebellar cortex, and brain stem. These findings suggest that age influences a broad network of brain regions, particularly those associated with memory and motor functions, with the cingulum playing a central role. Furthermore, although the network visually presents as two distinct subnetworks (left and right), both are connected through the corpus callosum. For the purposes of constructing a square/symmetric connectome, the corpus callosum (same as all other commissural fibers) was divided into left and right regions, but it is inherently a single region. This division is visual only as the interhemispheric connections, while not depicted inFigure 4, are confirmed by tractography shown inFigure 5, indicating that the two subnetworks are indeed interconnected.

**Fig. 5.**
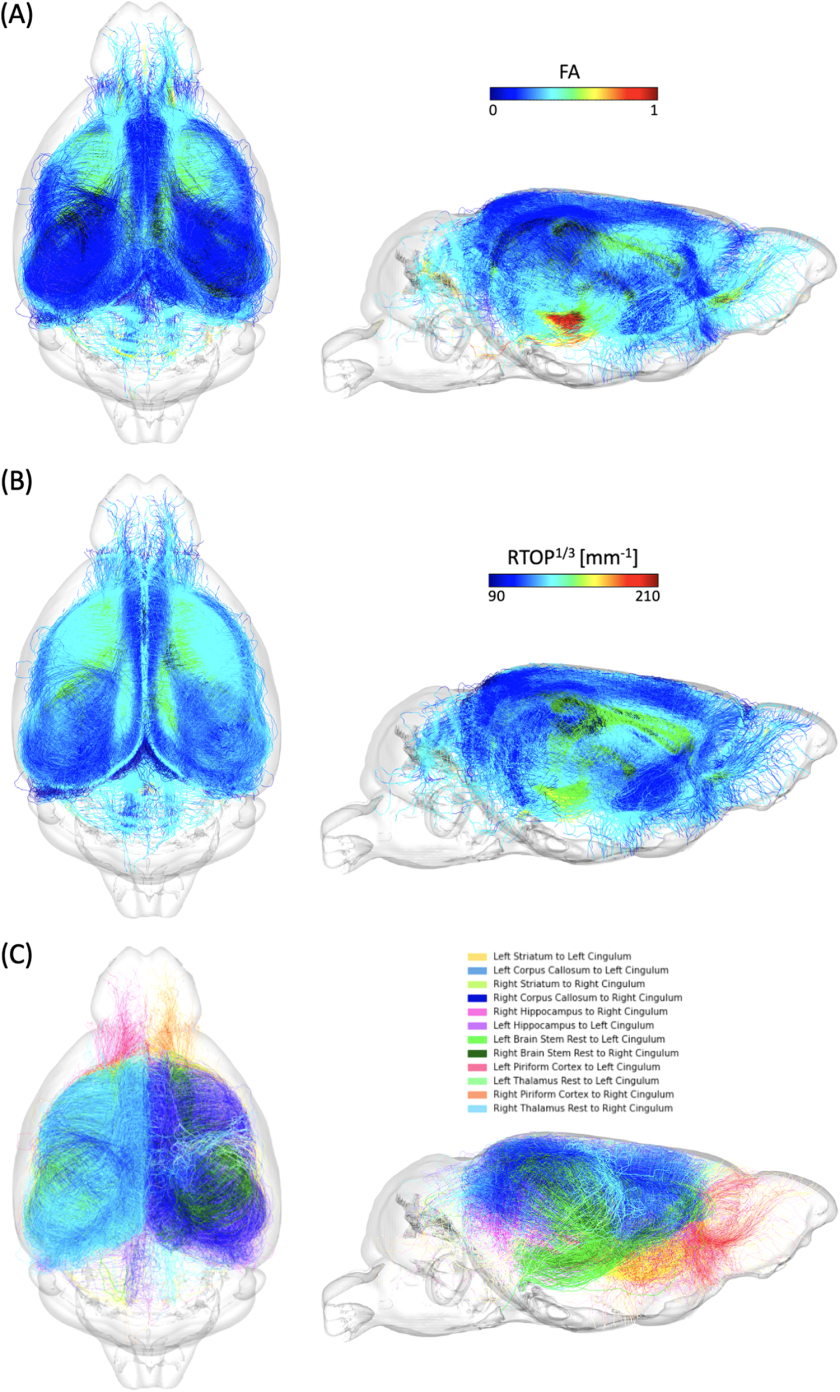
Visualization of the top 12 connections that contributed most to age prediction based on the quadrant attention module. (A) FA values along the corresponding tracts. (B) RTOP values along the corresponding tracts. (C) Illustration of the tractography with distinct colors for each connection.

To validate the output connections from the QAM, we performed a tractography analysis examining the FA and RTOP values across the top 12 tracts identified by the model (Fig. 5). The top 12 tracts include both left and right intrahemispheric connections between striatum, corpus callosum, hippocampus, brain stem, piriform cortex, and thalamus to cingulum. We visualized these tracts to show how they are spatially arranged, and the variability in FA and RTOP.

Tract-specific analyses were conducted across the two age groups (younger, old) for the top six tracts identified by FAGNN, which included cingulum to striatum, corpus callosum, and hippocampus for both left and right hemispheres. These analyses revealed how the brain’s structural integrity along tracts varies with aging, i.e., FA and RTOP decreased with age. The results, visualized inFigures 6and7, support the FAGNN model’s selections and offer insight into the microstructural properties of the tracts that may underpin age-related cognitive changes. Specifically, the FA analysis is shown inFigure 6and the RTOP analysis is shown inFigure 7. In both FA and RTOP figures, the general trend was similar, in that the striatum to cingulum connection and hippocampus to cingulum connection showed smaller differences between younger and old groups, but there was asymmetry between left and right hemispheric connection. The corpus callosum to cingulum tract comparison between younger and old revealed more significant differences between the two groups but a symmetrical trend for the left and right hemispheric connections.

**Fig. 6.**
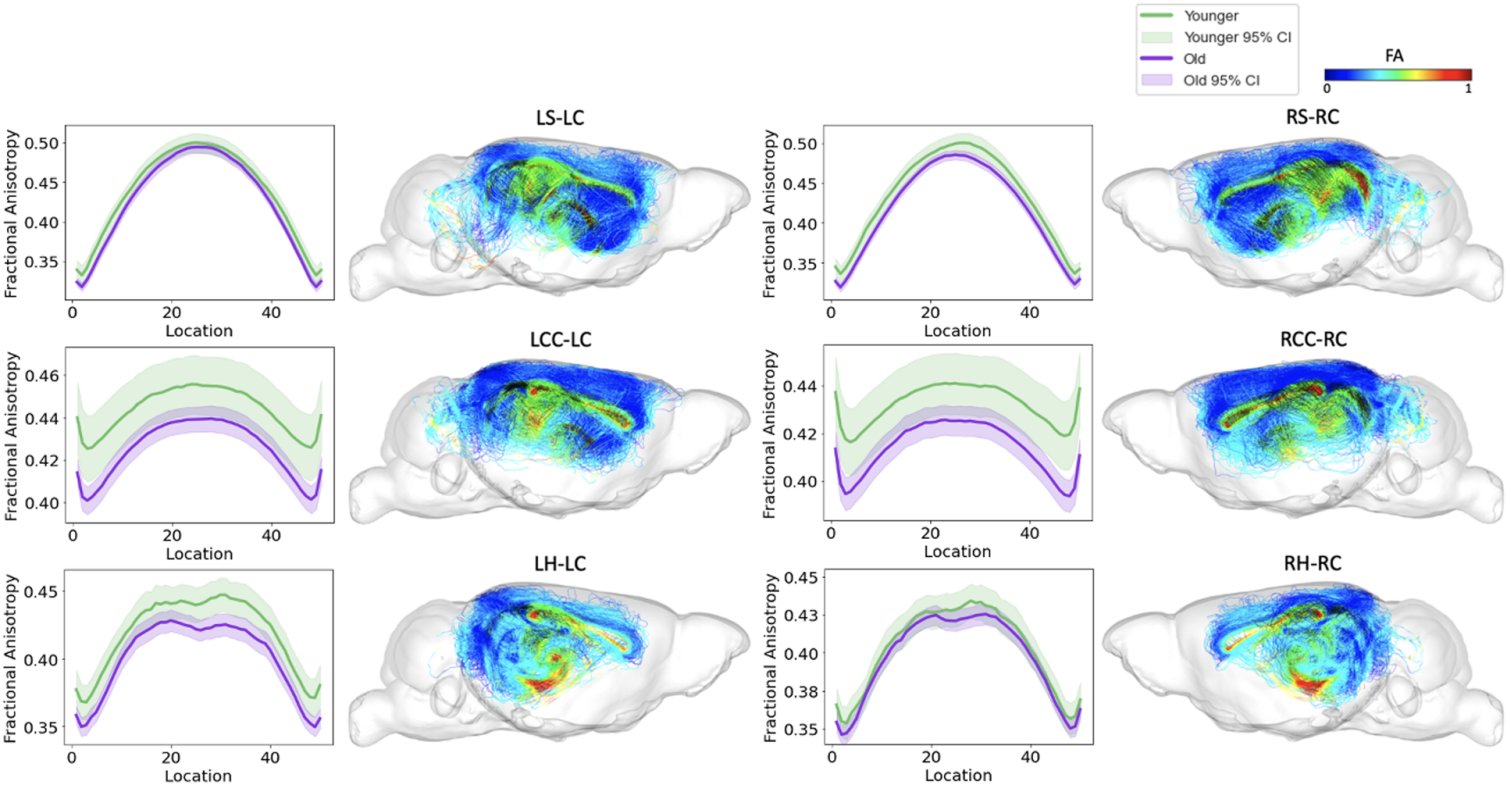
FA along tract profiles for the top six edges identified by FAGNN. The first row shows striatum-cingulum tract, second row shows corpus callosum-cingulum tract, and last row shows hippocampus-cingulum tract. First column shows FA profile of left-left connection, second column shows the corresponding tractography with FA values. Third column shows FA profile of right-right connection, and the last column shows the corresponding tractography with FA values. The FA values along each tract for age groups were significantly different with*p*< 0.01.

**Fig. 7.**
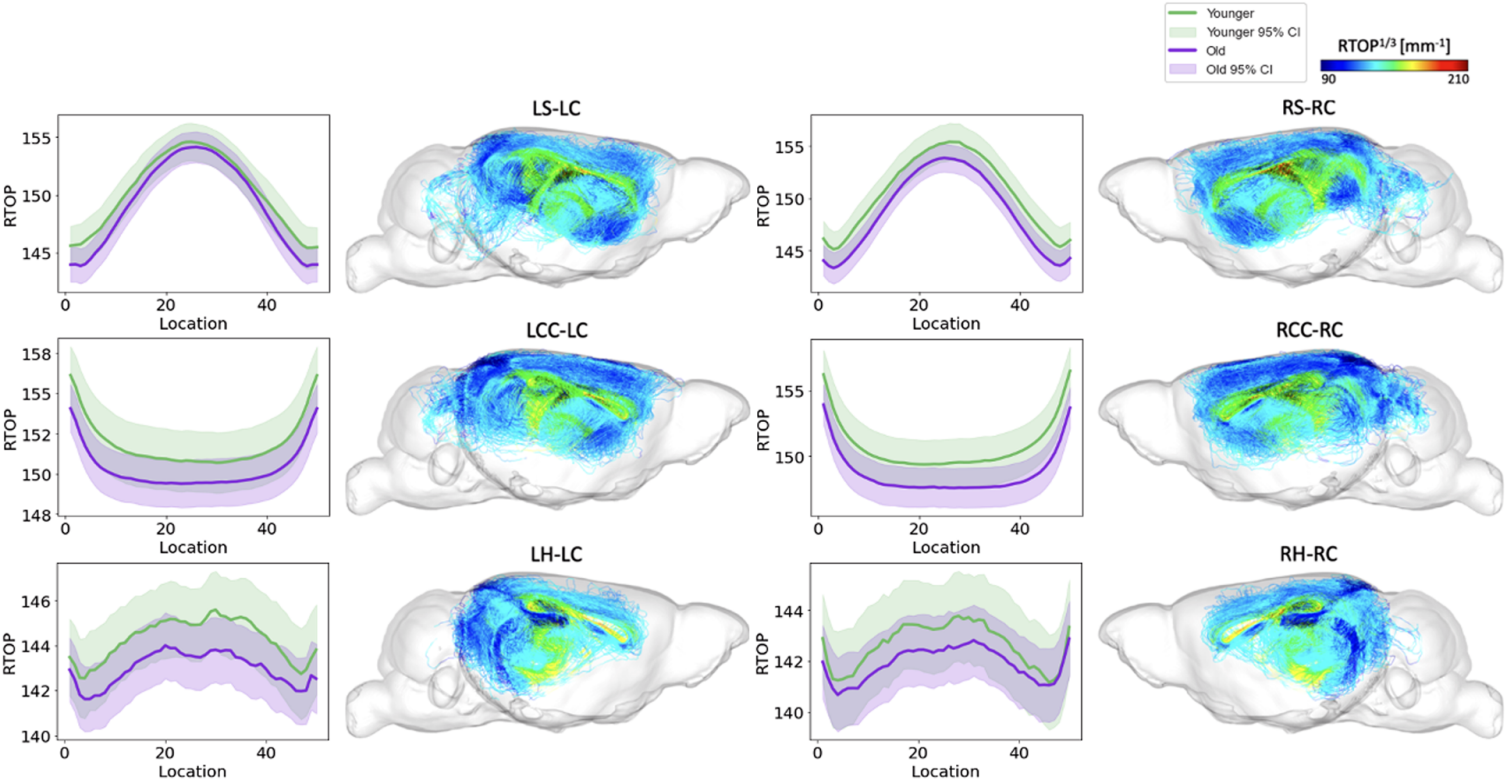
RTOP along tract profiles for the top six edges identified by FAGNN. The first row shows striatum-cingulum tract, second row shows corpus callosum-cingulum tract, and last row shows hippocampus-cingulum tract. First column shows RTOP profile of left-left connection, second column shows the corresponding tractography with RTOP values. Third column shows RTOP profile of right-right connection, and the last column shows the corresponding tractography with RTOP values. The RTOP values along each tract for age groups were all significantly different with*p*< 0.01.

SupplementaryFigures 1 and 2extend these analyses to include RTAP and RTPP values, further characterizing the microstructural details of these tracts and supporting the biological relevance of the connectomic features identified by the FAGNN model as markers of brain aging.

We further assessed the white matter integrity by examining the FA, RTOP, RTAP, and RTPP profiles of six randomly selected tracts not included among the top 20% highest edge scores identified by FAGNN. These profiles aimed to determine whether white matter decay, as indicated by DWI and MAP metrics, was prevalent across all brain areas with aging. As illustrated inSupplementary Figures 3–6, the metric comparisons between younger and old groups were generally similar. However, there were exceptions, such as in the FA profile from the left secondary motor cortex to the left dorsolateral orbital cortex (Supplementary Fig. 3A), where older subjects exhibited higher FA values, contrary to the trends observed in the top six connections. Other tracts, including connections from the left spinocerebellar tract to right cerebellar white matter, right insular cortex to left cingulate cortex area, right cerebral peduncle to left primary motor cortex, and right secondary auditory cortex ventral part to left hippocampus, displayed similar metrics across the age groups. The FA profile from the right cochlear nucleus to the right ventral tegmental area typically showed higher values across FA, RTOP, RTAP, and RTPP. While RTOP and RTAP profiles displayed a similar trend, RTOP profiles often highlighted more pronounced age-related differences, evident through qualitatively more vivid contrasts and lower*p*-values compared with RTAP. Nonetheless, RTPP values, as depicted inSupplementary Figure 6, consistently demonstrated that younger individuals had higher metrics, indicating a decline in RTPP values with aging across the brain. This suggests that RTOP, RTAP, and FA metrics are more sensitive to specific tracts that undergo changes with aging.

## 4 Discussion

The FAGNN introduced in this study represents a novel approach for predicting brain age from multivariate data (connectome, behavior, and traits) from mouse models of AD risk. To establish the efficacy of FAGNN, we conducted a comparative analysis with a modified BrainGNN (Li et al., 2021), which was originally designed for functional connectomes and for classification tasks, and that we adapted for structural connectomes and for continuous variable prediction tasks, i.e., age. We evaluated various configurations of our model that included distinct subnetworks to discern which combination and which subsets of the input data most effectively contribute to the accuracy of age prediction. We found that our full model, which integrates connectomes processed within a quadrant attention module, AD risk factor data, and behavioral metrics, outperformed all other models we have tested with MAE of 31.85 days and RMSE of 41.84 days. Further, FAGNN outperformed other models in sensitivity regarding the difference in MAE between control group and the risk factor group, as well as the difference in MAE from training and testing within the control groups and MAE from training on the control and testing on the risk factor groups, suggesting FAGNN’s both predicative accuracy and sensitivity to accelerated brain aging due to AD risk factors. The better performance of the comprehensive model emphasizes the importance of a multidimensional and multivariate approach, integrating diverse datasets for enhancing the model’s predictive accuracy and robustness.

Chronological age inherently influences a multitude of factors in mice, extending beyond those related to brain-related traits (De Magalhães et al., 2005;Kovacs et al., 2009). Thus, an accurate prediction of chronological age would necessitate the inclusion of many variables outside the scope of our dataset, which is primarily focused on factors pertinent to brain structural connectivity as a marker of successful or pathological aging. Thus, there is a disparity between the chronological age and the brain age predicted by our model, which is estimated based on cognition, AD-related traits, and brain connectivity, and that accelerated and delayed aging can be predicted using brain age prediction models (Wrigglesworth et al., 2021). We thus estimate the discrepancy between chronological and brain age as a marker of risk (Anatürk et al., 2021;Franke & Gaser, 2019). We have used the brain age delta between the chronological brain age and brain age, as estimated by the model (Cole et al., 2017;Grosenick et al., 2013;T. T. Le et al., 2018;Liang et al., 2019) to study accelerated or decelerated brain aging. A lower brain age delta could suggest that the brain’s connectomic structure with corresponding cognitive traits and AD risk factors are younger than its chronological equivalent, potentially indicating a protective mechanism against age-related changes. The variations in brain age delta observed in our study highlighted the influence of genetic and environmental factors and their interplay on the aging process of the brain.

The analysis of brain age delta between control and risk factor groups across two age categories (younger and old), as depicted inFigure 3, reveals sex as a significant differentiator within the younger group. Factors such as APOE genotype, diet, and human immune response genes were not significant in this group. In contrast, diet and human immune response gene significantly influenced brain age delta in the old group, supporting research linking immunity and dietary habits to neuroprotection and brain aging resilience (Jurgens & Johnson, 2012;Uranga et al., 2010). Surprisingly, APOE genotype did not significantly affect brain age delta. This may indicate that the humanized APOE response in mice requires interaction with other factors such as diet and immune responses to manifest significant aging-related changes. Future research should expand the sample size and include a broader age range to more thoroughly investigate the interplay between APOE genotype, diet, and immunity in brain aging. Further expansion to include young (6-12 months) and additional old mice would allow for a more even distribution across three age groups: young, middle aged, and old. Then, applying bias correction based on slope and intercept from regression of the control group could reveal more robust findings.

Our model identified the brain connections significantly contributing to brain age prediction, potentially mapping subnetworks vulnerable to aging and increased AD risk. The top 30 brain connections, determined by the quadrant attention module of the FAGNN model, are illustrated inFigure 4. These connections involve the piriform cortex, striatum, hippocampus, amygdala, thalamus, and hypothalamus, all converging on the cingulum. This selection aligns with previous studies highlighting the importance of these regions in aging (Bartsch & Wulff, 2015;Catheline et al., 2010;Fama & Sullivan, 2015;Shen et al., 2013;Swaab et al., 1992;Umegaki et al., 2008). Notably, these connections predominantly involved the cingulum, a white matter tract involved in a wide range of motivational and emotional behaviors, and importantly in spatial working memory (Schmahmann et al., 2007), as tested in the MWM portion of our study. The cingulum, running anterior to posterior, is considered part of the limbic circuit, and is highly integrated with other white matter tracts (Zerbi et al., 2013). It connects numerous regions and partakes in numerous functions related to memory, problem solving, attention, sensory, and regularization of heart rate and blood pressure (Gunbey et al., 2014). There have been previous studies that related cingulum to aging. A study found that sensory and motor functions, which are associated with the cingulum, decline with aging and are reflected by changes in the integrity of the white matter tracts, including the cingulum (Madden et al., 2012). The top 30 connections linked either the left or right cingulum to other regions, supporting the important role of the cingulum and its subnetworks in aging, and in relation to AD risk (Bennett et al., 2015;Gold et al., 2012). Our results suggest that additional smaller tracts connecting various brain regions to the cingulum play a significant role as well. Previous studies have shown demyelination of anterior-posterior white matter tracts with aging (Madden et al., 2012;Sibilia et al., 2017;Sullivan & Pfefferbaum, 2006). These previous studies motivate further studies of white matter integrity and myelination with aging, expanding from tracts such as cingulum to its connections, as identified by the FAGNN model.

The model assigned edge scores to connections based on prediction accuracy, and connections with higher edge scores more significantly influenced the network’s age prediction decisions. These selections are complex and not immediately intuitive, which further prompted investigation into the basis of the network’s choices. We aimed to validate the network’s findings by examining tractography features of the structural connectome, utilizing diffusion and MAP metrics related to the white matter integrity of the tractography in the brain (Avram et al., 2016;Kochunov et al., 2012;Pitteri et al., 2021;Zucchelli et al., 2016). The tracts with FA and RTOP values are depicted inFigure 5A and B. Previous studies have found decreases in diffusion metrics—namely FA values in DTI—in the cingulum with aging (Catheline et al., 2010;Jang et al., 2016;Sibilia et al., 2017). Also, cognitive control in older adults is sensitive to variations in the cingulum microstructure, particularly for subjects with MCI (Metzler-Baddeley et al., 2012). In mice, there was a decrease in myelinated fiber length density and FA in the cingulum bundle with aging, regardless of genotype, hemisphere, or sex (Segal et al., 2010). In our investigation, we have expanded the study of white matter integrity using MAP properties including RTOP, RTAP, and RTPP. Although the importance of cingulum has been stressed in previous studies, there is sparse information on the properties of its connections to other regions, especially in mice. We expanded the information on the properties of the cingulum connections to specific regions such as striatum, corpus callosum, and hippocampus. This was made possible by high resolution, ex vivo multishell diffusion MRI studies, and advances in accelerated acquisitions that benefit from compressed sensing (Anderson et al., 2018;N. Wang et al., 2018). Additionally, the techniques were sensitive to diffusion properties (Anderson et al., 2020), which allowed for a more detailed representation of tracts and increased sensitivity in detecting significant connections using deep learning models.

We concentrated on the top six connections identified by the model for aging, examining the tract profiles of streamlines within these specific connections, including striatum to cingulum, corpus callosum to cingulum, and hippocampus to cingulum, for both left-to-left and right-to-right hemispheric connections. This focus was to uncover the underlying information of the connectivity and validate the model’s selections. The tract profiles were examined to discern metric values along the tracts, differentiating the white matter integrity and characteristics across the age groups in our dataset, categorized as younger and old mice with age cutoff at 15 months. InFigure 6, we observed a significant age-related decrease in FA values, with the higher values in younger group and lower values in old group. This aligns with expectations and is consistent with previous research on the cingulum (Catheline et al., 2010;Jang et al., 2016;Segal et al., 2010;Sibilia et al., 2017).Figure 7displays a similar trend in RTOP values, showing highly significant difference between the age groups. Supplementary Figures 1 and 2 present RTAP and RTPP values along the tracts, respectively. The general trend for FA, RTOP, RTAP, and RTPP values across age groups was consistent: younger subjects exhibited the higher values and old subjects showed the lower values. Notable changes in FA, RTOP, RTAP, and RTPP values within these tracts denote significant variations in the microstructural integrity of the brain tracts. A decrease in RTOP, RTAP, and RTPP values indicates neuronal density reduction, and possibly axonal swelling (H. Le et al., 2020). These findings were anticipated, as we expected these tracts to deteriorate with aging. This validation underscored the neuroanatomical relevance of the connectome features identified by the FAGNN model as markers of brain aging and emphasized white matter integrity as a key factor in aging and neurodegeneration. The top six connections displayed either asymmetry between left-left and right-right connections or significant differences across the two age groups. Specifically, connections from the striatum to cingulum and hippocampus to cingulum showed asymmetry between the left and right intrahemispheric connections. Furthermore, the corpus callosum demonstrated substantial differences between younger and old groups, indicating that the model could assess both symmetry across hemispheric connections and the degree of white matter degradation, as verified by FA and MAP metrics. Although previous studies support the inference about the symmetry of white matter tracts, the specificity of these findings in the context of aging remains unclear and warrants further investigation to validate these observations (Honnedevasthana Arun et al., 2021;Roe et al., 2023;Yeatman et al., 2014).

The potential impact of our study consists in showing proof-of-principle that integrating the FAGNN model with clinical data may advance the early detection of AD, enhance the precision of disease staging, and tailor intervention strategies more effectively. The FAGNN model could prove helpful for tracking the progression of brain-related diseases and assessing the effectiveness of treatments over time. Moreover, the specific tracts identified in this study that connect the cingulum to other important regions require further validation through larger studies. Future studies that enhance the integration of various modalities could significantly advance AD research.

Nonetheless, our study is not without limitations. Transitioning from rodent models to human applications is an important but difficult procedure (Casey et al., 2015;Pappas & Nagy, 2019), which would benefit from cross-species studies. The results of this study could be tested through longitudinal human studies for subjects with different AD risk factors. Our results suggest that expanding the model with more comprehensive data, e.g., from omics studies, could help improve prediction accuracy and robustness, thereby yielding more reliable results (Mahood et al., 2020;Miotto et al., 2017), and refining predictive accuracy. We need to consider the higher heterogeneity of human subjects who may contribute to brain aging, and that using large public databases on human aging such as UK Biobank (Sudlow et al., 2015) can help refine models, while mouse data are scarce. We recognize that the sample size of our current study is modest for deep learning training, especially in comparison with human datasets, but the genetic heterogeneity is also reduced and the environment in which these mice were raised was controlled. Our model’s inclusion of multiple datasets and the consequent increase in the number of parameters could complicate the model training. Also, as we included four risk factors, as well as continuous age for prediction, there are thus numerous permutations of various risk factor data, and the distribution of age range of mice for each group is not perfectly balanced. The skewed age distribution across the two groups, 12-15 months and 15-20 months, posed challenges in the training process due to the absence of very young mice (<12 months) and a limited number of old mice. Despite these challenges, our results illustrate that incorporating risk factors and behavioral data into the model enhances the accuracy and precision of predictions, even as the model increases in size and complexity.

In conclusion, our research presents a comprehensive model for predicting brain age, incorporating a diverse set of data, from structural connectomes to environmental, genetic, and cognitive factors. We have revealed subnetworks that correlate to brain aging, and that multivariate data integration is beneficial for predictive modeling. These efforts represent a significant stride toward a more personalized medicine in managing neurodegenerative diseases, and pave the way for future studies aimed at validating and broadening the application of connectomic biomarkers for aging and age-associated neurodegenerative diseases, such as AD.

## Supplementary Material

Supplementary Material

## Data Availability

The raw connectome, behavioral data, and associated metadata are available athttps://zenodo.org/records/10372075. The code necessary to reproduce the original analyses is available fromhttps://github.com/hsmoon0/FAGNN.
