# Supplementary material for: Feature attention graph neural network for estimating brain age and identifying important neural connections in mouse models of genetic risk for Alzheimer’s disease
Source: Imaging Neurosci (Camb). 2024 Jul 31;2:imag-2-00245. doi: 10.1162/imag_a_00245 (PMC12272265; doi:10.1162/imag_a_00245)
Supplement: Supplementary Material [file imag_a_00245-supp.pdf]

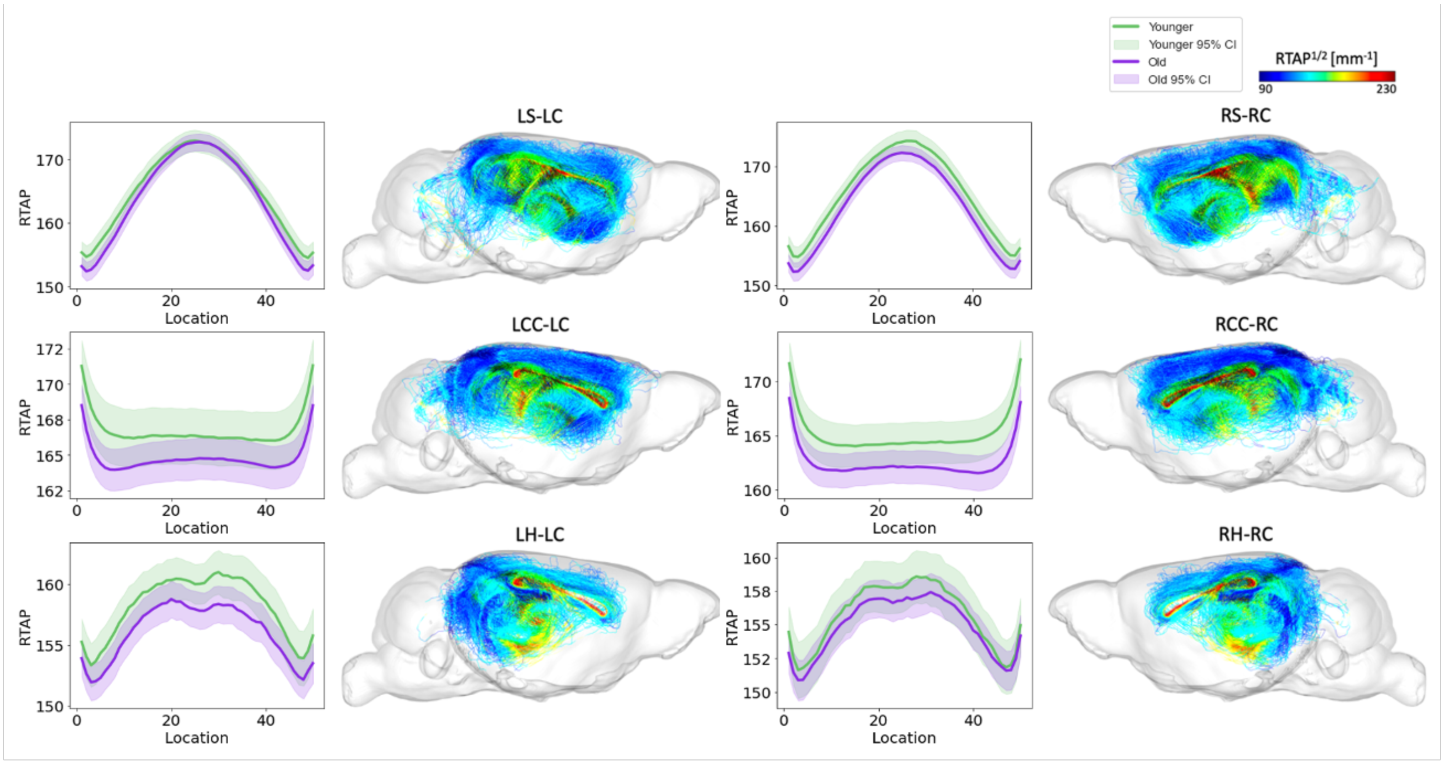

Supplementary Figure 1. RTAP along tract profiles for the top 6 edges identified by FAGNN. The first row shows striatum-cingulum tract, second row shows corpus callosum-cingulum tract and last row shows hippocampus-cingulum tract. First column shows RTAP profile of left-left connection, second column shows the corresponding tractography with RTAP values. Third column shows RTAP profile of right-right connection, and the last column shows the corresponding tractography with RTAP values. The RTAP values along each tract for age groups were all significantly different with $p < 0.001$.

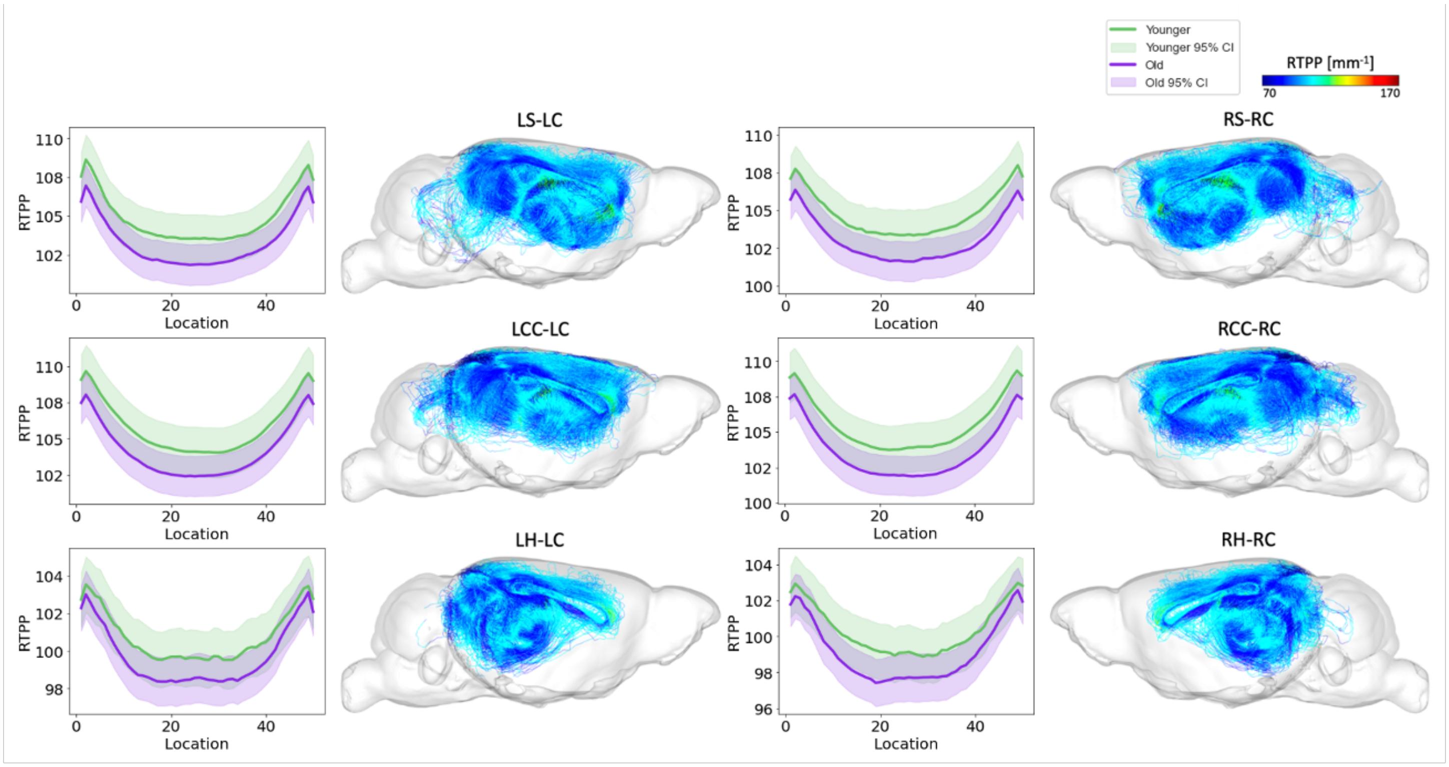

Supplementary Figure 2. RTPP along tract profiles for the top 6 edges identified by FAGNN. The first row shows striatum-cingulum tract, second row shows corpus callosum-cingulum tract and last row shows hippocampus-cingulum tract. First

column shows RTPP profile of left-left connection, second column shows the corresponding tractography with RTPP values. Third column shows RTPP profile of right-right connection, and the last column shows the corresponding tractography with RTPP values. The RTPP values along each tract for age groups were all significantly different with $p < 0.001$.

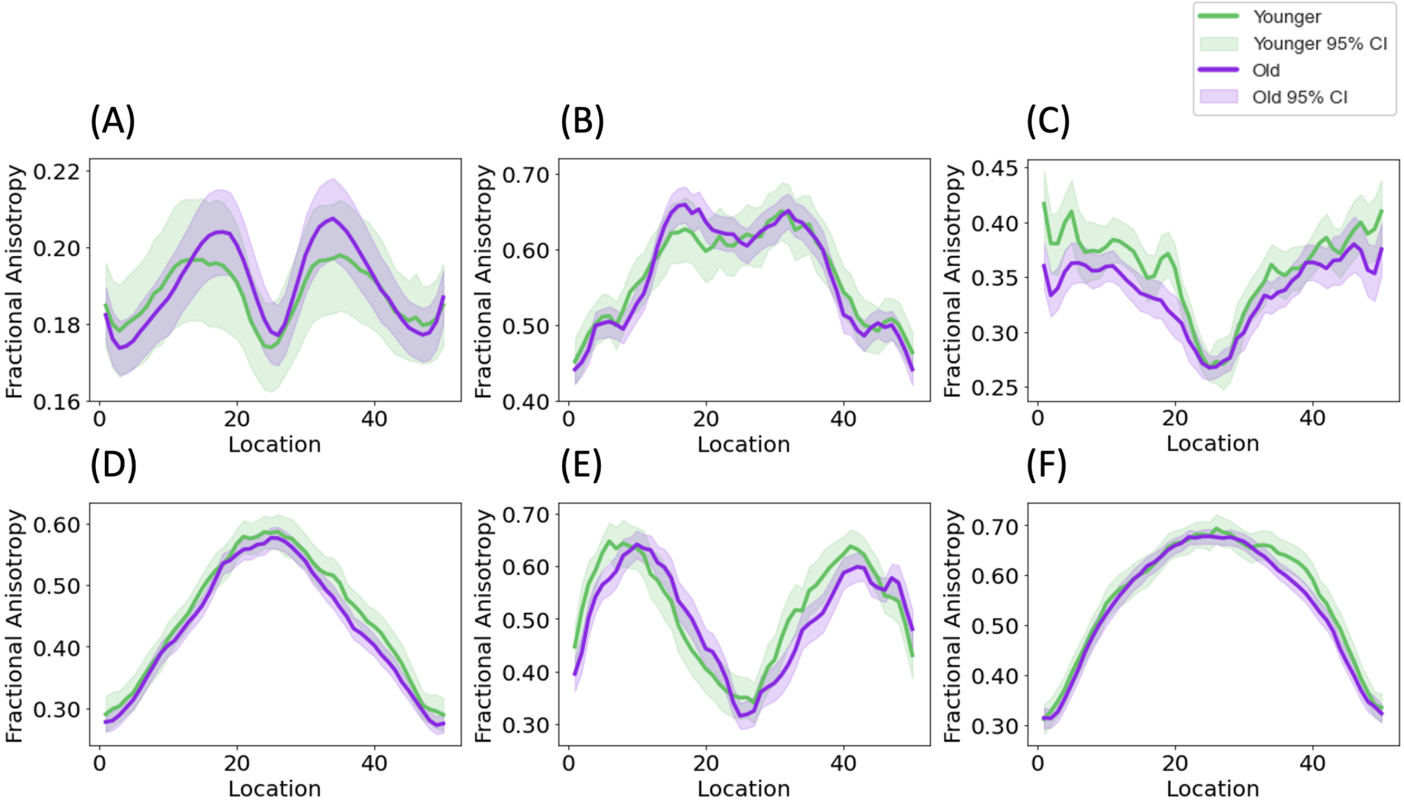

Supplementary Figure 3. FA along tract profiles for random 6 edges identified by FAGNN that are outside top 20%. (A) left striatum to left ventral thalamic nuclei (B) left corpus callosum to left ventral thalamic nuclei (C) left hippocampus to left ventral thalamic nuclei (D) right striatum to right ventral thalamic nuclei (E) right corpus callosum to right ventral thalamic nuclei (F) right hippocampus to right ventral thalamic nuclei.

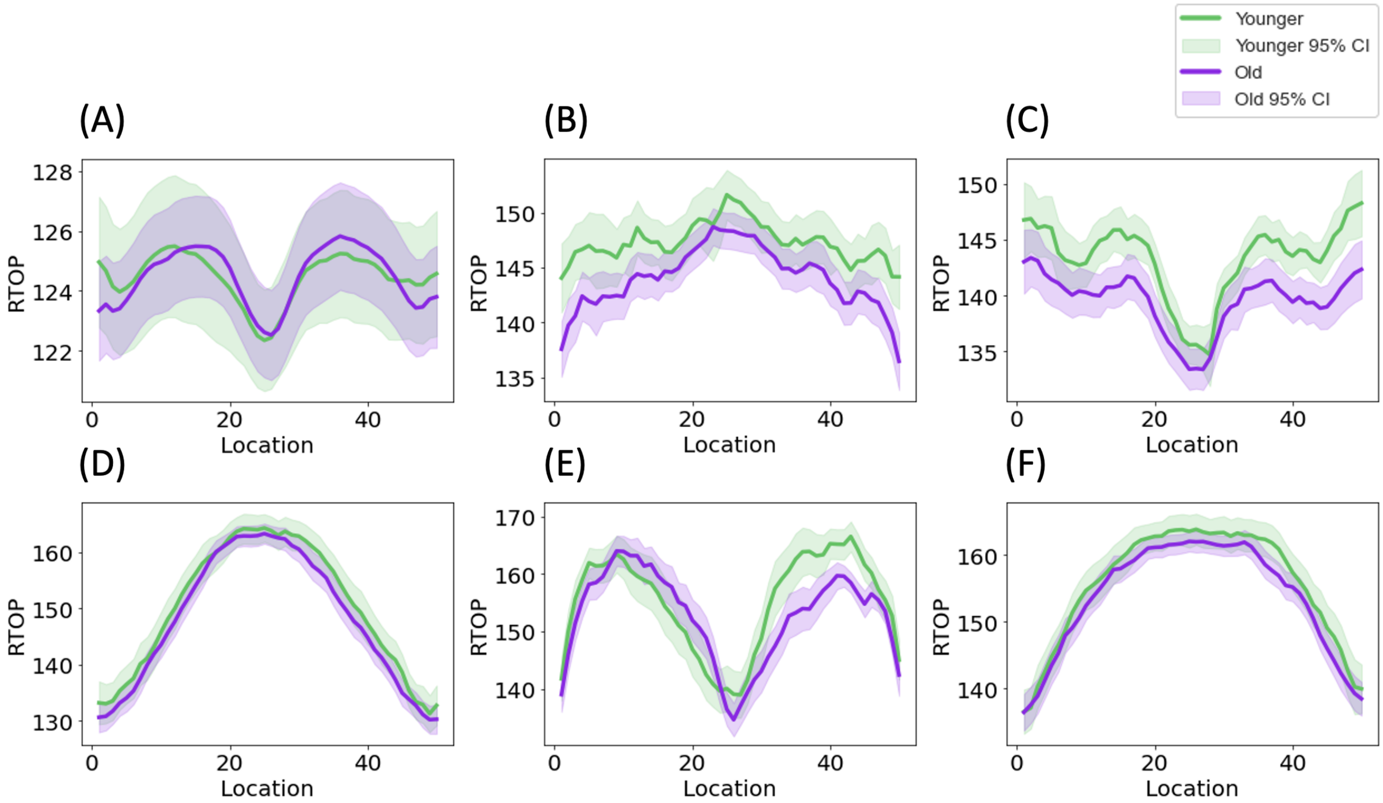

Supplementary Figure 4. RTOP along tract profiles for random 6 edges identified by FAGNN that are outside top 20%. (A) left striatum to left ventral thalamic nuclei (B) left corpus callosum to left ventral thalamic nuclei (C) left hippocampus to left ventral thalamic nuclei (D) right striatum to right ventral thalamic nuclei (E) right corpus callosum to right ventral thalamic nuclei (F) right hippocampus to right ventral thalamic nuclei.

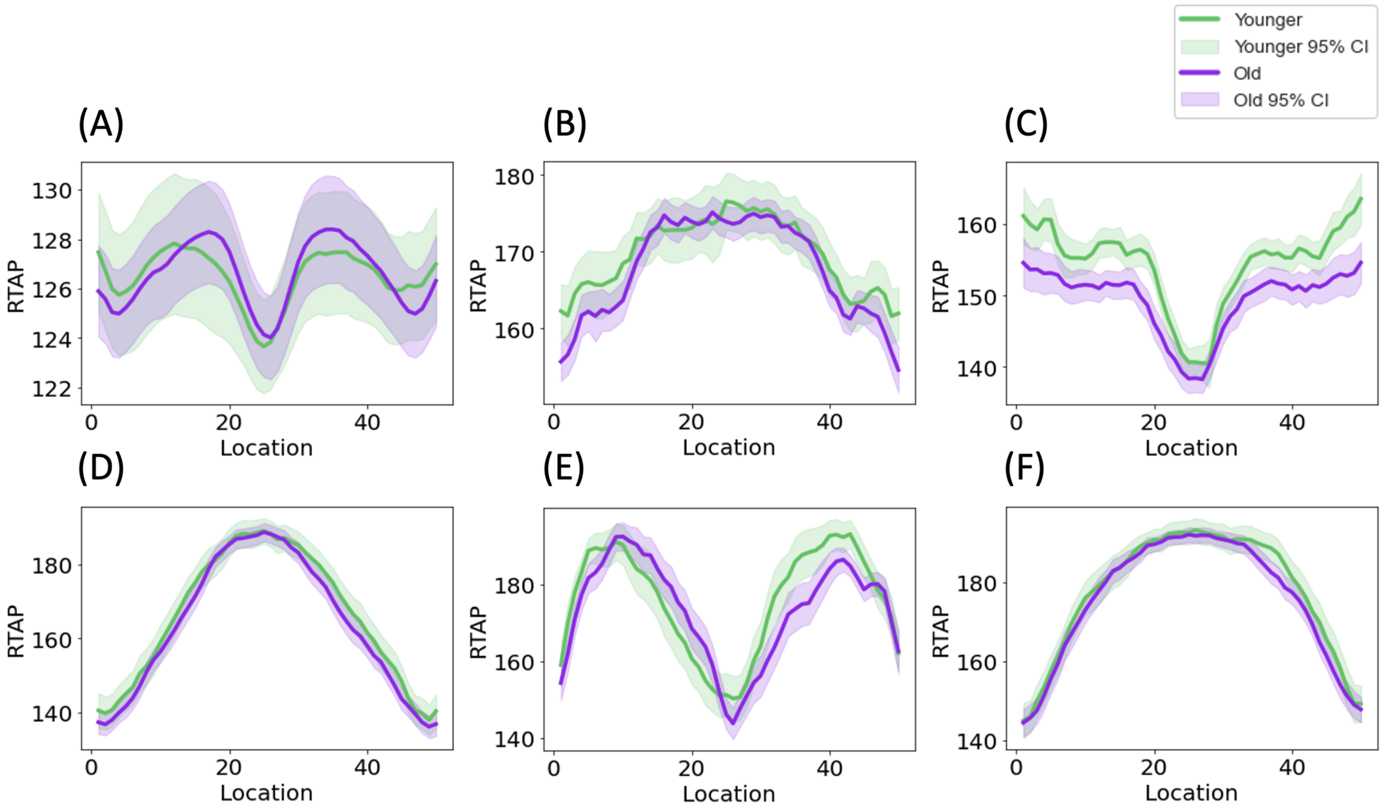

Supplementary Figure 5. RTAP along tract profiles for random 6 edges identified by FAGNN that are outside top 20%. (A) left striatum to left ventral thalamic nuclei (B) left corpus callosum to left ventral thalamic nuclei (C) left hippocampus to left ventral thalamic nuclei (D) right striatum to right ventral thalamic nuclei (E) right corpus callosum to right ventral thalamic nuclei (F) right hippocampus to right ventral thalamic nuclei.

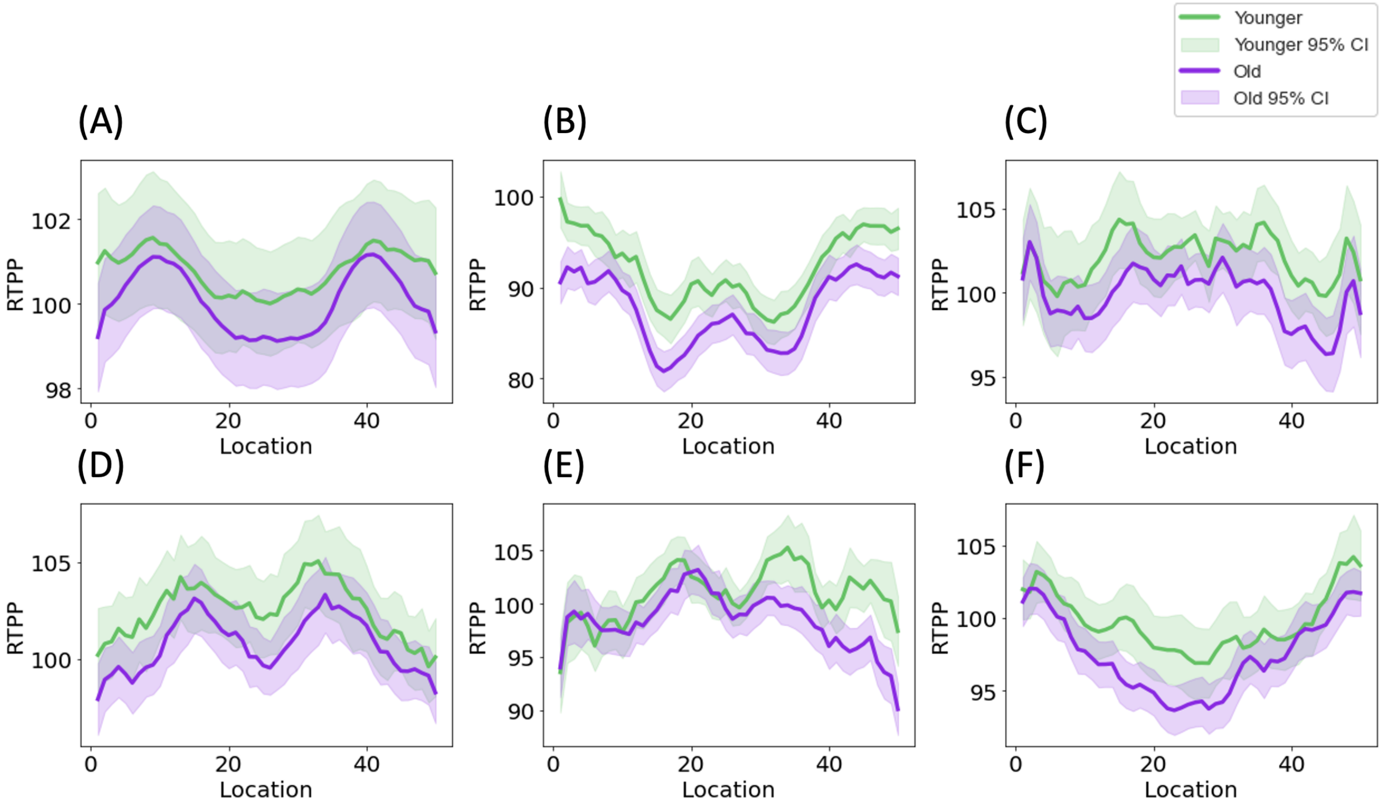

Supplementary Figure 6. RTPP along tract profiles for random 6 edges identified by FAGNN that are outside top 20%. (A) left striatum to left ventral thalamic nuclei (B) left corpus callosum to left ventral thalamic nuclei (C) left hippocampus to left ventral thalamic nuclei (D) right striatum to right ventral thalamic nuclei (E) right corpus callosum to right ventral thalamic nuclei (F) right hippocampus to right ventral thalamic nuclei.
